# Pharmacological Inhibition of Spermine Oxidase Suppresses Excitotoxicity Induced Neuroinflammation in Mouse Retina

**DOI:** 10.3390/ijms23042133

**Published:** 2022-02-15

**Authors:** Moaddey Alfarhan, Fang Liu, Shengshuai Shan, Prahalathan Pichavaram, Payaningal R. Somanath, S. Priya Narayanan

**Affiliations:** 1 Clinical and Experimental Therapeutics Program, Department of Clinical and Administrative Pharmacy, University of Georgia, Augusta, GA 30912, USA; malfarhan@augusta.edu (M.A.); fliu1@augusta.edu (F.L.); sshan@augusta.edu (S.S.); sshenoy@augusta.edu (P.R.S.); 2 Research Division, Charlie Norwood VA Medical Center, Augusta, GA 30901, USA; 3 Vision Discovery Institute, Augusta University, Augusta, GA 30912, USA; prahalathan.pichavaram@stjude.org; 4 Department of Clinical Pharmacy, College of Pharmacy, Jazan University, Jazan 45142, Saudi Arabia

**Keywords:** neuroinflammation, polyamine oxidation, spermine oxidase, acrolein, microglia, oxidative damage, antioxidant signaling

## Abstract

Polyamine oxidation plays a major role in neurodegenerative diseases. Previous studies from our laboratory demonstrated that spermine oxidase (SMOX, a member of the polyamine oxidase family) inhibition using MDL 72527 reduced neurodegeneration in models of retinal excitotoxicity and diabetic retinopathy. However, the mechanisms behind the neuroprotection offered by SMOX inhibition are not completely studied. Utilizing the experimental model of retinal excitotoxicity, the present study determined the impact of SMOX blockade in retinal neuroinflammation. Our results demonstrated upregulation in the number of cells positive for Iba-1 (ionized calcium-binding adaptor molecule 1), CD (Cluster Differentiation) 68, and CD16/32 in excitotoxicity-induced retinas, while MDL 72527 treatment reduced these changes, along with increases in the number of cells positive for Arginase1 and CD206. When retinal excitotoxicity upregulated several pro-inflammatory genes, MDL 72527 treatment reduced many of them and increased anti-inflammatory genes. Furthermore, SMOX inhibition upregulated antioxidant signaling (indicated by elevated Nrf2 and HO-1 levels) and reduced protein-conjugated acrolein in excitotoxic retinas. In vitro studies using C8-B4 cells showed changes in cellular morphology and increased reactive oxygen species formation in response to acrolein (a product of SMOX activity) treatment. Overall, our findings indicate that the inhibition SMOX pathway reduced neuroinflammation and upregulated antioxidant signaling in the retina.

## 1. Introduction

Neuroinflammation is an important cause in the development of neurodegenerative diseases [1,2]. Long-term inflammation exposure causes cellular damage [3]. Studies show that inflammation has an essential role in the pathogenesis of several ocular diseases, including age-related macular degeneration (AMD) [4], diabetic retinopathy [5], and retinitis pigmentosa [6]. Injured neurons can release damage-associated molecular patterns (DAMPs), and these DAMPs can bind to their receptors on microglial cells and cause microglia activation [7]. Activation of microglia, which is the primary resident immune cell in the brain and retina, results in the release of inflammatory mediators such as cytokines, reactive oxygen species (ROS), and nitric oxide (NO) [8]. Retinal excitotoxicity is suggested as a major mechanism of neurodegeneration, and experimental models of excitotoxicity are extensively used for studying retinal ganglion cells (RGCs) damage and dysfunction in ocular diseases, including optic neuropathy [9,10], glaucoma [11,12], and diabetic retinopathy [13,14].

Polyamines including spermine, spermidine, and putrescine are important for cellular functions such as proliferation, differentiation, and apoptosis [15]. N1-acetylpolyamine oxidase (APAO) and spermine oxidase (SMOX) regulate polyamine catabolism, but the activation of these enzymes causes the release of H_2_O_2_ and acrolein [16]. While SMOX is a highly inducible enzyme, APAO is constitutively expressed [15]. Acrolein has been shown to induce inflammation in mouse BV2 microglial cells and function as a neurotoxin causing increased cell death in a rat model [17,18]. N1N4-bis (2, 3-butadienyl)-1,4-butane diamine (MDL 72527) is selective-irreversible inhibitor for APAO and SMOX [19,20,21]. Previous studies have reported that MDL 72527 treatment showed neuroprotective effects in ischemic brain injury in rats by reducing edema formation and reduction in ischemic injury volume [22,23]. Treatment with MDL 72527 was also neuroprotective against edema formation and necrotic formation in rats after traumatic brain injury [24].

Earlier studies from our laboratory have demonstrated that MDL 72527 treatment reduced retinal neurovascular damages in hyperoxia models [25] and retinal excitotoxicity [26]. Studies performed on the experimental model of DR demonstrated that the inhibition of SMOX by treatment with MDL 72527 improved RGC survival, retinal structure, and function in diabetic mice [27]. Our previous study has also reported an increase in the expression of SMOX during retinal excitotoxicity and the degeneration of neurons, while MDL 72527 treatment improved neuronal survival [26]. However, the mechanisms behind the neuroprotective effects of SMOX blockade are not studied. Neuroinflammation is considered a major cause of neurodegeneration, and hence using a mouse model of retinal excitotoxicity, the present study was undertaken to investigate whether the inhibition of SMOX by MDL 72527 can ameliorate neuroinflammation.

## 2. Results

### 2.1. SMOX Inhibition Using MDL 72527 Reduced Iba-1 Positive Cells in the Excitotoxic Retina

**Figure 1:**
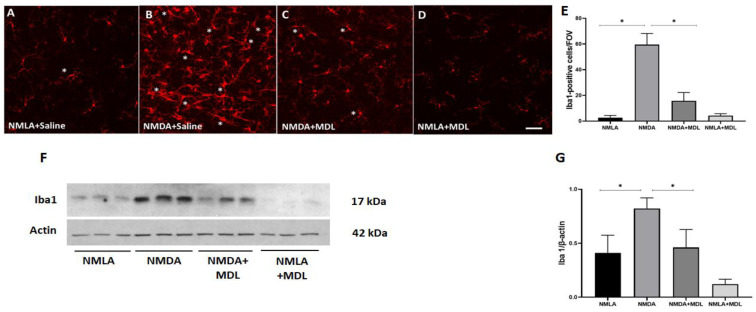
Treatment with MDL 72527 reduced Iba-1 positive cells with activated morphology in the excitotoxic retina. (**A**–**D**) Immunofluorescence staining of retinal flat mounts using Iba-1 antibody and representative confocal images presented. Asterisks indicate retinal microglia presenting activated morphology (7 days post-injury). (**E**) Bar graph representing the quantification studies of Iba-1 positive cells with activated morphology (N = 4–6 per group, * *p* < 0.01). (**F**,**G**) Results of Western blot studies show the increased protein levels of Iba-1 in NMDA retinas compared to NMLA controls. Iba-1 expression was reduced in response to MDL 72527 treatment. * *p* < 0.01, N = 4–5 per group. Scale bar 50 µm. Results presented as Mean ± SD.

Our previous study has shown that SMOX blockade by MDL 72527 significantly improved neuronal survival in the excitotoxic retinas [26]. In the present study, we first investigated the status of the inflammatory cells positive for Iba-1 (ionized calcium-binding adaptor molecule 1), a marker specific for microglia/macrophages. Immunofluorescence staining of retinal flatmounts demonstrated an increase in the number of Iba-1 positive cells presenting activated morphology in the retinas from mice treated with NMDA (7 days post-injury) compared to their NMLA controls (Figure 1A,B). In response to SMOX inhibition by treatment with MDL 72527, a marked reduction of Iba-1 positive microglia/macrophage cells was observed in comparison with the NMDA group (Figure 1B,C). MDL 72527 treatment did not result in any noticeable changes in the control retina (Figure 1D). Quantification studies indicated a significant increase in cells with activated morphology, while MDL 72527 treatment significantly reduced this effect (Figure 1E). Furthermore, our immunoblot studies using retinal lysates showed that the upregulation in the expression of Iba-1 protein level in NMDA retinas was significantly reduced in response to MDL 72527 treatment (Figure 1F,G). Altogether, these results suggest the potential of MDL 72527 treatment in reducing inflammation in excitotoxic retinas.

### 2.2. Impact of MDL 72527 Treatment on the Status of Inflammatory Cells in Response to Excitotoxicity

**Figure 2:**
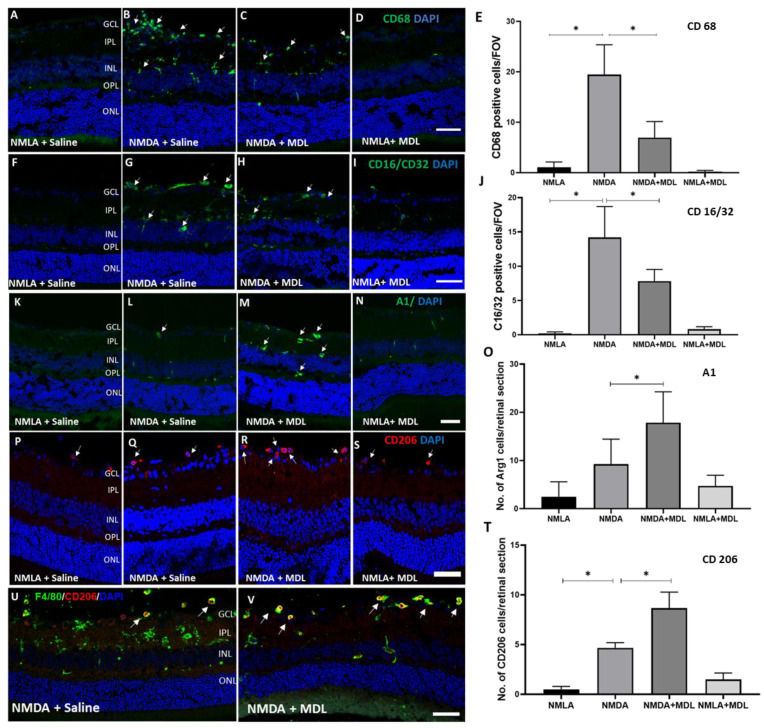
SMOX inhibition altered the level of M1 and M2 microglia/macrophage phenotypes in the excitotoxic retinas. Representative confocal images of retinal cryostat sections immunostained using CD 68 (**A**–**D**), CD16/32 (**F**–**I**), A1 (**K**–**N**), and CD206 (**P**–**S**). Bar graphs showing the quantification studies demonstrate that number of cells positive for M1 markers, CD 68 (**E**), and CD 16/32 (**J**) are upregulated in excitotoxic retinas while MDL 72527 significantly downregulated the changes. Quantification of data showed a significant upregulation in the number of cells positive for M2 markers, A1(**O**), and CD 206 (**T**) in the NMDA retinas in response to MDL 72527 treatment. (**U**,**V**) Confocal images of retinal sections immunostained using F 4/80 and CD 206. Arrows represent the colocalization of the two markers. The number of animals used N = 3–6 per group. * *p* < 0.05, 3 days post-injury. Scale bar 50 μm. Arrows indicate representative cells positive for the markers used. Results presented as Mean ± SD.

We next investigated whether SMOX inhibition using MDL 72527 affected different populations of inflammatory cells, earlier to neuronal damage. Microglia/macrophages in the CNS have been reported to adopt distinctive phenotypes, including the classically activated (M1) state and the alternatively activated (M2) state in response to various stimulations [28]. Immunofluorescence staining of retinal cryostat sections (3 days post-injury) was performed using markers of M1/M2 phenotypes of microglia/macrophages. Our results show that the number of M1 phenotype cells positive for CD (Cluster of Differentiation) 68 and CD 16/32 were significantly increased in excitotoxicity-induced retinas (Figure 2A,B,F,G). This effect was significantly reduced in the excitotoxic retinas in response to the treatment with MDL 72527 (Figure 2C,H). On the contrary, studies using M2 markers showed significant upregulation in the cells positive for Arginase (A) 1 and CD 206 in MDL 72527 treated NMDA retinas compared to the vehicle-treated group (Figure 2L,M,Q,R). Our immunofluorescence studies indicated that most of the CD 206 positive cells are colocalized with cells positive for F4/80. MDL 72527 treatment did not cause any marked changes in the status of CD68 or CD16/32 positive cells in the control groups. However, the slight upregulation observed in A1 and CD206 in the NMLA control group in response to SMOX inhibition was not significant. These results indicate that SMOX blockade can reduce inflammatory cells in response to excitotoxicity.

### 2.3. Effect of SMOX Inhibition on Pro- and Anti-Inflammatory Cytokines

**Figure 3:**
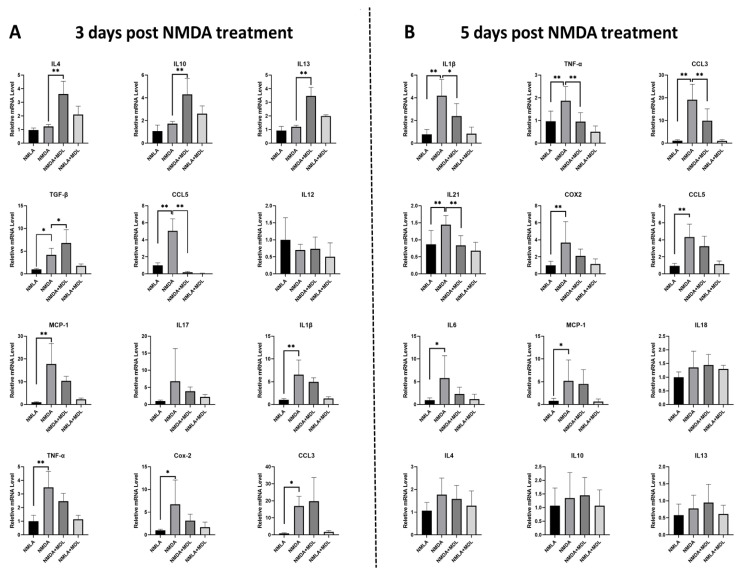
Effect of SMOX blockade on cytokines levels in the excitotoxic retinas. Quantitative RT-PCR analysis demonstrating changes in mRNA levels of pro- and anti-inflammatory cytokines and chemokines in excitotoxic retinas following 3 and 5 days post-injury. (**A**) Following 3 days post-injury, in comparison with the vehicle-treated group, MDL 72527 treated excitotoxic retinas showed significant upregulation in the mRNA levels of anti-inflammatory cytokines (IL-4, IL-10, IL-13, and TGFβ) and reduced level in the pro-inflammatory CCL5. (**B**) Following 5 days post-injury, the upregulated levels of IL-1β, TNFα, CCL3, and IL-21 mRNA levels were significantly reduced by MDL 72527 treatment in the NMDA groups. The upregulation observed in COX2 and CCL5 is not significantly reduced by SMOX inhibition. IL-6 and MCP-1 were also significantly upregulated in NMDA retinas. The mRNA levels of IL-18, IL-4, IL-10, and IL-13 did not indicate significant changes in any groups. Data are presented as mean ± SD. ** *p* < 0.01; * *p* < 0.05. N = 5–8 per group.

We next investigated the changes in the status of inflammatory molecules in relation to SMOX blockade (Figure 3). Analysis of mRNA levels using qRT-PCR at two different time points (3 and 5 days) following excitotoxic insult was performed (Figure 3A,B). Our results indicated that, in response to MDL 72527 treatment, there is an early upregulation in the anti-inflammatory cytokines followed by downregulation of pro-inflammatory molecules in the excitotoxic retinas. Significant upregulation of many pro-inflammatory genes including interleukin (IL-) 1β, IL-21, C-C motif chemokine ligand (CCL) 5, CCL3, cyclooxygenase 2 (COX2), tumor necrosis factor-alpha (TNF-α), and chemoattractant protein-1 (MCP-1) in NMDA retinas was observed at both time points as compared to the control retinas. As evidenced in Figure 3A, 3 days post-injury, MDL 72527 treatment significantly upregulated many anti-inflammatory genes IL-4, IL-10, IL-13, and transforming growth factor β (TGF-β) in the excitotoxic retinas, CCL5 is the only pro-inflammatory gene that showed a significant downregulation. MDL 72527 treatment did not reduce the mRNA levels of other pro-inflammatory molecules (Figure 3A). Interestingly, on day 5 following excitotoxicity, SMOX inhibition demonstrated reduced levels of several of the inflammatory molecules such as IL-1β, CCL3, IL-21, and TNF-α levels in NMDA retinas. However, the levels of anti-inflammatory genes were similar across the two NMDA treated groups at this time point (Figure 3B). The decrease observed in the levels of IL-6, COX2, CCL5, and MCP-1 were, however, not statistically significant (Figure 3B). These results further suggest the potential of SMOX blockade in reducing the inflammatory signals in response to retinal excitotoxicity.

### 2.4. MDL 72527 Treatment Activated Antioxidant Signaling in the Excitotoxic Retinas

**Figure 4:**
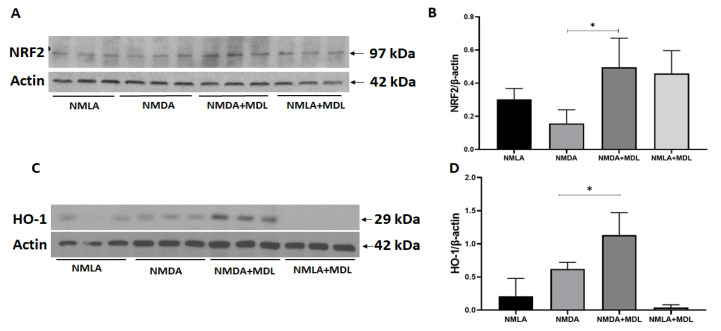
Blockade of SMOX by treatment with MDL 72527 increased antioxidant signaling. (**A**,**B**) Western blot studies and quantification data showed a significant increase in Nrf2 expression in MDL 72527 treated excitotoxic retinas compared to the vehicle-treated NMDA group. (**C**,**D**) Results show that MDL 72527 treatment significantly increased the expression of HO-1 expression in excitotoxic retinas in comparison with the vehicle-treated group. * *p* < 0.05, N = 5–6 per group and representative blots are presented. Results presented as Mean ± SD.

In the present study, we further investigated if the inhibition of SMOX impacts the antioxidant signaling pathway. As shown in Figure 4, immunoblotting experiments indicated that the blockade of SMOX with MDL 72527 significantly increased the expression of Nrf2 (nuclear factor erythroid 2-related factor 2) in the NMDA retinas compared to the vehicle-treated group (Figure 4A,B). Further studies on the expression of heme oxygenase-1 (HO-1), a target gene for Nrf2, indicated that the inhibition of SMOX using MDL 72527 significantly upregulated the expression of HO-1 in the NMDA retinas in comparison to the vehicle-treated NMDA retinas (Figure 4C,D). The changes observed in Nrf2 or HO-1 in NMDA retinas compared to the respective vehicle controls were not significant. These results suggest the potential of MDL 72527 in activating the antioxidant signaling in response to retinal injury.

### 2.5. Changes in Acrolein Conjugated Proteins in the Excitotoxic Retinas

**Figure 5:**
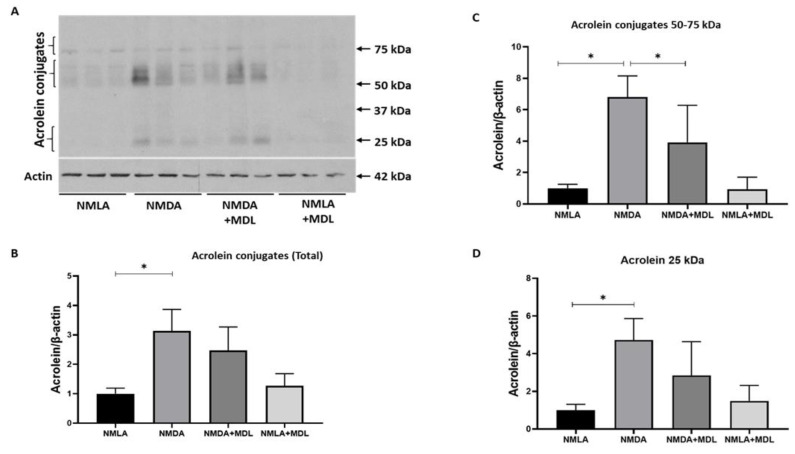
Impact of SMOX inhibition on acrolein conjugated proteins. (**A**) Western blot analysis showing the expression of acrolein-conjugated proteins in NMDA treated retinas. (**B**–**D**) Quantification data showing the marked upregulation of acrolein conjugated proteins at various molecular weight ranges and the effect of MDL 72527 treatment. * *p* < 0.05, N = 5–6 per group and representative blots are presented. Results presented as Mean ± SD.

Acrolein, a highly reactive aldehyde, directly results from SMOX activation [29] and can elevate oxidative damage by conjugating with proteins, lipids, and DNA [30,31]. In the present study, we studied the levels of protein-conjugated acrolein in NMDA retinas and the impact of SMOX inhibition on their distribution (Figure 5A). A significant increase in the level of protein conjugated acrolein was observed in NMDA retinas when compared to the NMLA control group, and the treatment with MDL 72527 reduced acrolein conjugated proteins to some extent (Figure 5A). MDL 72527 markedly reduced acrolein conjugated proteins at a molecular weight around 50–60 kDa (Figure 5B). However, the downregulation observed in the total conjugated proteins and at molecular weight ranges around 25 kDa was not significant in response to MDL 72527 treatment.

### 2.6. Impact of Acrolein Treatment on Microglia Cells In-Vitro

**Figure 6:**
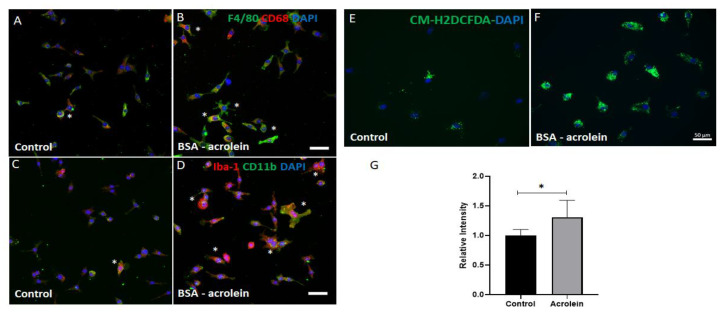
Impact of BSA-conjugated acrolein treatment on microglia cells in-vitro. (**A**–**D**) Confocal images for C8-B4 cells immunostained using F4/80, CD68, Iba-1, and CD11b antibodies showing changes in morphology in response to BSA-acrolein treatment (6 h treatment with 25 μg/mL BSA-acrolein). (**E**,**F**) Images showing the increased ROS levels in C8-B4 cells, studied by H2DCFDA assay and the quantification of ROS levels analyzed by Image J (**G**). Experiments were repeated a minimum of 3 times, and representative images are presented. * *p* < 0.05. Scale bar = 50 μm. Results presented as Mean ± SD.

Acrolein is a mediator for oxidative stress [32] and microglia are the primary source for ROS formation in the central nervous system [33]. Using C8-B4 cells with microglial properties, in the current study, we investigated the effect of bovine serum albumin (BSA)-conjugated acrolein on the morphological changes and the formation of reactive oxygen species on microglia. As shown in Figure 6, treatment of C8-B4 cells with 25 μg/mL of BSA-conjugated acrolein for 6 h altered the cellular morphology. Immunostaining using markers CD 11b, F4/80, CD 68, and Iba-1 showed changes such as an increase in size and thick processes (Figure 6A–D) in the treated group. H2DCFDA assay showed elevated ROS formation in BSA-acrolein-treated cells compared to the control group (Figure 6E,F). Quantification of fluorescence intensity showed significantly upregulated ROS levels in response to acrolein treatment (Figure 6G). These results suggest that SMOX activation could lead to microglial activation and ROS formation.

## 3. Discussion

The current study provides the first specific evidence for the impact of inhibiting SMOX pharmacologically, using MDL 72527, in limiting neuroinflammation in a mouse model of retinal excitotoxicity. Retinal excitotoxicity is recognized as a mechanism of neurodegeneration associated with ocular diseases, including DR [34]. Previous studies from our laboratory have shown that the SMOX expression is upregulated in the retinal neurons in models of neurovascular injuries, and the increase in expression was accompanied by a reduction in neuronal survival, retinal function, and changes in retinal structure [26,27,35]. Inhibition of SMOX with MDL 72527 showed protective effects against these changes [26,27,35]. However, the molecular mechanisms underlying the neuroprotective effects of SMOX inhibition are not completely understood. Neuroinflammation is a major cause of neuronal damage, and in the present study, we are demonstrating the effect of MDL 72527 on ameliorating inflammation and oxidative stress in a mouse model of retinal excitotoxicity. To the best of our knowledge, this is the first report investigating the impact of SMOX inhibition in excitotoxicity-induced neuroinflammation in the retina. 

SMOX is primarily expressed in neurons in the brain [36,37,38] and retina [25,26,27]. A previous study has shown the localization of SMOX in glial cells of fibrovascular tissue from PDR patients [39]. However, the overactivation of SMOX has its effects on other cell types. The reactive aldehydes and H_2_O_2_ generated as byproducts of SMOX activation can impact other cells such as glia and endothelial cells [35,40,41,42,43,44,45]. In the recent study by Fan et al. using the rat model of cerebral Ischemia/Reperfusion, the authors demonstrated that neuron-derived SMOX induction after stroke was essential for microglial activation and inflammation [37]. Previously, Cervelli et al. showed that neuronal overexpression of SMOX induced microglial activation in the aged neocortex [38]. Targeting SMOX by treatment with MDL 72527 demonstrated neuroprotective effects in rats with ischemic brain damage [22] and traumatic brain injury [24]. A previous study has shown that downregulation of SMOX decreased infarction volume, neurological deficit, and reduced neuronal apoptosis and inflammation [37]. Studies from our laboratory have indicated that treatment with MDL 72527 also protected retinal neurons in models of oxygen-induced retinopathy (OIR) [25] and diabetic retinopathy [27]. Another study utilizing SMOX-deficient mice showed a reduction in *H. pylori*-induced inflammation [46]. Similar effects were also noticed when treated with MDL 72527 [47]. Enterotoxigenic Bacteroides fragilis-induced chronic inflammation in C57BL/6 mice was also shown to be reduced by MDL 72527 treatment [48]. However, not many studies have investigated the impact of SMOX inhibition on inflammation associated with retinal neuronal damage and dysfunction. 

Studies have shown that NMDA treatment results in increased loss of RGCs, and intraocular NMDA injection is useful to study RGCs degeneration in retinal diseases [49,50,51,52,53]. The NMDA receptor is a type of glutamate receptor in the mammalian central nervous system [54]. In the retina, NMDA receptors are expressed in both neurons and Müller glia [53,55]. While NMDA receptor activation causes neuronal damage in the retina [56], Müller cells are reported to undergo proliferation [57,58]. Hypertrophy of Müller glia is also a characteristic feature observed in response to excitotoxicity [26,59]. In the diabetic retina, the level of glutamate is increased due to Müller cell dysfunction, which is responsible for glutamate metabolism in the retina [60]. Glutamate level is also reported to be elevated in the vitreous of glaucoma and diabetic patients [61,62,63]. Accumulation of glutamate and other excitatory molecules activates NMDA receptors, increases calcium influx, and causes neuronal cell death due to excessive stimulation [64]. The most affected cells are in the inner retinal layers, including ganglion cells, amacrine cells, and bipolar cells [26,65]. Intravitreal injection NMDA induced macrophages/microglia infiltration and increased chemoattractant protein-1 (MCP-1) production in addition to an increase of IL-1β expression [66]. Consistent with these results, our present study demonstrates that the number of activated microglia/macrophages and pro-inflammatory cytokines are increased in the excitotoxic retinas. However, these changes are reversed in response to MDL 72527 treatment, supporting the impact of SMOX inhibition on reducing inflammation. An earlier study reported from our lab, using the OIR model of neurovascular damage, has also shown suppression of microglia/macrophages with activated morphology and downregulation of inflammatory cytokines in response to MDL 72527 treatment [35].

Inflammation plays an important role in developing ocular diseases such as DR [67], AMD [4], retinitis pigmentosa [6], and glaucoma [68]. Neuroinflammation is characterized by sustained activation of glial cells such as microglia and astrocyte and the recruitment of other immune cells [69]. Microglia represent the resident immune cells in the retina, and it is important to maintain retinal homeostasis; however, they are associated with the progression of diseases such as AMD [70], DR [71], and glaucoma [72]. Microglia in normal retina exhibit ramified morphology but in response to injury, microglia exhibit amoeboid morphology and increase the migration and proliferation to the injury’s site [73]. In response to various stimuli/insults, the microglia could adopt distinctive phenotypes, including the classically activated (M1) state or the alternatively activated (M2) state. The M1-like phenotype is characterized by increased expression of surface markers such as CD16/32, CD68, CD40 and the production of pro-inflammatory mediators including IL-1β, TNF-α, and IL-6 and thus accelerating the inflammatory process [74]. Alternatively, microglia could assume an M2 phenotype, releasing anti-inflammatory, protective and trophic factors, including IL-4, IL-10, 1L-13, and TGF-β, and triggering anti-inflammatory responses [75,76]. Activated microglia and macrophages secrete pro-inflammatory mediators (cytokines, chemokines, etc.) and upregulate the expression of inflammatory mediators such as inducible nitric oxide synthase and can act on other cells, including neurons [77]. Our findings show an increase in the number of cells positive for CD68 and CD16/32 in response to excitotoxicity, but the treatment with MDL 72527 reversed these effects and upregulated the number of cells in the M2 phenotype. Consistent with these observations, our mRNA results show an early upregulation in anti-inflammatory cytokines followed by a downregulation in pro-inflammatory molecules. RGCs are severely affected in retinal excitotoxicity models. Interestingly, our study shows that most of the CD 206 positive cells are localized at the GCL of the MDL treated excitotoxic retina, suggesting the possible role of MDL 72527 in the recruitment of M2 microglia/macrophages for RGC survival. Studies have also shown that activated microglia can send signals to Müller cells and cause changes in Müller cells’ morphology and function [78]. Reactive Müller cells can also produce pro-inflammatory factors, which in turn can increase microglial activation and migration. These bidirectional feedback signals between these two cell types are meant to restore retinal homeostasis in case of injury or disease [79]. The previous study showed that SMOX inhibition reduced Müller glial injury, as studied by changes in GFAP expression [26].

Acrolein is a toxic aldehyde produced during the oxidation of polyamines [80]. Acrolein can bind with proteins causing their dysfunction [81]. In our study, a significant increase in acrolein conjugated proteins was observed, and this was reduced to some extent by MDL 72527 treatment. Further studies are required to identify the specific modifications caused by acrolein. Acrolein has been shown to activate microglia in the CNS [82,83], induce proinflammatory signals, and increase cell migration of microglial cells [17]. However, in addition to acrolein formation, SMOX activation can lead to the formation of H_2_O_2_ and thus further elevating oxidative stress [16]. Our previous study showed the activation of primary microglia in response to H_2_O_2_, another byproduct of SMOX activity [35]. Besides its role in producing inflammatory cytokines, microglia is also considered a major producer of ROS in CNS [33]. Consistent with these, our study showed acrolein altered morphology and elevated ROS formation in C8-B4 cells. Furthermore, an earlier study showed that the treatment of microglial cells treated with supernatant from SMOX-downregulated neurons following OGD/R showed less activation and reduced IL-6 and TNF-α [37].

Oxidative stress and inflammation are closely related pathophysiological processes. Agents with antioxidant and anti-inflammatory properties would be an attractive therapeutic strategy. In the present study, we investigated if the inhibition of SMOX has an impact on antioxidants signals. The stimulation of NMDA receptors by administration of NMDA affects the antioxidants status [84]. The nuclear factor erythroid 2-related factor 2 (Nrf2) plays an important role in regulating several antioxidant enzymes [85], and its activation protects the retina from retinal diseases [86]. Nrf2 expression protected against oxidative stress in retinal diseases such as DR [87] and AMD [88]. Our study found that the treatment with MDL 72527 increased the expression of Nrf2 and its downstream target HO-1 regulated by Nrf2. HO-1 is an inducible protein in response to different stimuli, and its expression is reported to be increased as a defense in the retina in cases such as diabetic retinopathy [89], ischemia-reperfusion [90], and light-induced damage [91]. Induction of HO-1 expression showed a protective effect on RGCs in diabetic rats by reducing inflammation, apoptosis, and proliferation effects in the diabetic retina [89]. Induction of HO-1 expression also reduced the retinal damage against IR injury [92]. Taken together, MDL 72527 treatment reduced the production of acrolein and reduced the activation of the microglial cell.

**Figure 7:**
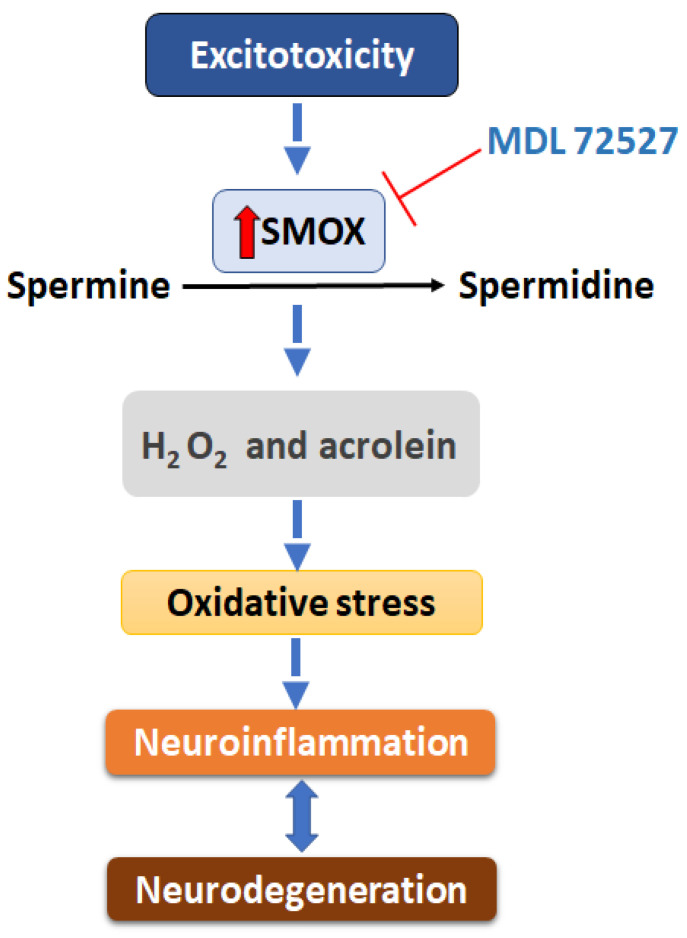
Proposed mechanism of excitotoxicity-induced neuronal damage. A diagrammatic representation of the proposed molecular mechanism of SMOX mediated neuroinflammation and neurodegeneration in the retina.

In summary, the inhibition of SMOX by the treatment with MDL 72527 reduced microglia/macrophage activation, induced changes in polarization status, downregulated proinflammatory cytokines release, decreased acrolein formation, and activated the antioxidant signals in the model of retinal excitotoxicity. A proposal of SMOX mediated neuroinflammation and neurodegeneration in the retina is depicted in Figure 7. Our studies report for the first time the specific effect of SMOX inhibition, using MDL 72527 treatment, in limiting neuroinflammation in the retina. We have demonstrated a crucial role for the SMOX signaling pathway as one of the major mechanisms associated with inflammation and neurodegeneration in the excitotoxic retina. Considering the need for new therapies for patients suffering from ocular diseases associated with retinal neuroinflammation, such as diabetic retinopathy or glaucoma, our results are highly relevant from a clinical perspective. Our findings suggest that targeting SMOX signaling can be considered a therapeutic strategy to treat neurodegenerative diseases of the eye.

## 4. Materials and Methods

### 4.1. Animals

All the procedures using animals were performed by the Association for Research in Vision and Ophthalmology (ARVO) Statement for the Use of Animals in Ophthalmic and Vision Research and were approved by the institutional animal care and use committee (Animal Welfare Assurance no. A3307–01) adhered to the Public Health Service Policy on Humane Care and Use of Laboratory Animals (revised July 2018). The protocols are approved by the Institutional Animal Care and Use Committee of the Augusta University (2016-0823) and the Charlie Norwood VA Medical Center, Augusta (18-11-110). Wild-type male C57BL6J mice (8–10 weeks, Jackson Laboratories, Bar Harbor, ME) were used in this study, and efforts were made to assure the minimum possible suffering during experimental procedures. 

### 4.2. Induction of Retinal Excitotoxicity

Retinal excitotoxicity was induced according to the method established in our laboratory [26]. Mice were anesthetized with 73 mg/kg ketamine hydrochloride and 7.3 mg/kg xylazine hydrochloride intraperitoneally. Pupils were dilated with 1% tropicamide (Akorn, Lake Forest, IL, USA). One drop of proparacaine hydrochloride (Akorn) was applied to the cornea as topical anesthesia. NMDA (N-Methyl-D-Aspartate, (Sigma, St. Louis, MO, USA), 20 n moles/eye, 1 µL, dissolved in saline) was injected intravitreally into the right eye using a beveled 35 G needle (NF35BV-2, World Precision Instruments, Sarasota, FL, USA) connected with an SGE Syringe (World Precision Instruments, Sarasota, FL, USA). The needle was moved out slowly after maintaining for 30 s. Antibiotic ointment was applied to prevent infection. NMLA (N-Methyl-L-Aspartate, 20 n moles/left eye) was used as control.

### 4.3. MDL 72527 Treatment

Treatment with polyamine oxidase inhibitor MDL 72527 (40 mg/kg/day, Sigma) in 0.9% saline was performed as described [26]. Treatment started one day prior to NMDA or NMLA injection and continued until the end of the respective experiments (varied from three to seven days). Normal saline was used as vehicle treatment.

### 4.4. Immunostaining of Retinal Flat-Mount and Quantification of Iba-1 Positive Cells

Immunostaining of retinal flat mounts was performed as described previously [35,93]. Eyes were removed and fixed in 4% paraformaldehyde overnight at 4 °C. After removing the cornea, sclera, lens, vitreous, and hyaloid vessels, four radial incisions were made to allow flattening of the retina. Retinas were permeabilized with 10% Triton X-100 for 20 min and blocked with 10% normal goat serum containing 1% BSA and 0.1% Triton X-100 for 1 h at room temperature. Retinas were stained with Iba-1 (Table 1) at 37 °C for 2 h, followed by secondary antibody Alexa-Fluor 555 goat anti-rabbit (1:500) (Life Technologies, Carlsbad, CA, USA) overnight at 4 °C. Retinas were washed three times in PBS and flat-mounted in mounting medium (Vectashield; Vector Laboratories, Burlingame, CA, USA). Flatmounts were examined using a confocal microscope (Zeiss LSM 510 META, Thornwood, NY, USA) and serial images were acquired. Quantification of Iba-1 positive cells (with activated morphology) GCL layer was performed using NIH Image J software as described in the previous reports from our laboratory [26,94,95]. Images (non-overlapping fields from the optic nerve to edge) were collected from each quadrant of the flat-mounted retina at the mid periphery (defined as halfway between the optic nerve head and the outer periphery). Five serial images (1 μm apart) of Iba-1 positive cells were taken from each region and merged to get a projection image for quantification. Cells showing activated morphology were selected manually (using point tool) and quantified using the analyzed particle function of Image J v1.53k.

### 4.5. Western Blotting

Retinal tissues were collected, snap-frozen immediately, and stored at −80 °C until used. Samples were homogenized in 1X RIPA lysis buffer (Millipore, Billerica, MA, USA) containing protease inhibitor cocktail (Thermo Scientific™) and phosphatase inhibitor cocktail (Thermo Scientific™). Protein concentration was estimated using BCA Protein Assay Kit (Thermo Scientific™). Approximately 20–30 ug of protein samples were separated on 8–12% SDS-PAGE gel, transferred to PVDF membrane, and blocked in 5% non-fat dry milk in 1X Tris-buffered saline with 0.1% Tween (TBST) for 1 h. Membranes were incubated overnight at 4 °C with respective primary antibodies (Table 1). The membranes were then washed with TBST 3 times and incubated with respective secondary antibodies (anti-rabbit or anti-mouse HRP-conjugated secondary antibody, Table 1) for 1 h at room temperature. The membranes were washed with TBST 3 times. Protein bands were detected using enhanced chemiluminescence (ECL) (Thermo Fisher). Band intensities were quantified using ImageJ software and normalized to beta-actin as the loading control.

### 4.6. Immunofluorescence Staining and Quantification of Cells on Retinal Sections

Immunostaining of retinal cryostat sections was performed as per the methods established in our laboratory [27,93,96]. Briefly, eyes were removed and fixed in 4% paraformaldehyde overnight at 4 °C and cryoprotected in 30% sucrose for 24 h. Cryostat sections (10 μm) obtained on glass slides were permeabilized in 0.1% Triton X-100 (30 min) followed by blocking in 10% normal donkey serum for 1 h at room temperature. Sections were incubated with primary antibodies (Table 1) overnight at 4 °C. Next day, the sections were washed 3 times with PBS and then incubated with Fluorescein-conjugated secondary antibodies (Table 1) for 2 h at room temperature. The sections were then washed 3 times in PBS and covered with a mounting medium with DAPI (Vectashield; Vector Laboratories, Burlingame, CA, USA). Images were obtained using a Zeiss (Thornwood, NY, USA) Axioplan Imager microscope and Zeiss Axiovision 4.8.2 software. Images (40X) were taken at 500 µm from the optic nerve, and the number of cells positive for CD68 or CD16/32 was counted manually. To quantify A1 or CD206 positive cells, counting was performed on retinal sections manually from the optic nerve head to the periphery. A minimum of three sections (20 µm apart) were used per animal, and a minimum of five animals were used per group.

### 4.7. RNA Isolation and Quantitative RT-PCR

The total mouse retina was homogenized by a Micro-Tube homogenizer (F65100-0000, SP BEL-ART) using QIAzol Lysis Reagent (79306, Qiagen, Hilden, Germany). Total RNA from homogenized retinal tissues was extracted using miRNeasy mini kit (217084, Qiagen). The concentration of RNA was measured using a Nanodrop Lite Spectrophotometer (ND-LITE-PR, Thermo Fisher Scientific, Waltham, MA, USA). Around 500 ng of total RNA was used for cDNA synthesis using a High-Capacity cDNA Reverse Transcription Kit (4368814, Applied Biosystems, Waltham, MA, USA). Quantitative PCR was carried out by StepOnePlus™ Real-Time PCR System (4376600, Applied Biosystems) using Power SYBR Green Master Mix (4309155, Applied Biosystems). Sequences of primers used in this study are listed in Table 2. Data were normalized to hypoxanthine phosphoribosyltransferase (HPRT), and the fold change between levels of different transcripts was calculated by the ΔΔCT method relating to this study.

### 4.8. Cell Culture and Conjugated Acrolein Treatment and Immunocytochemistry

C8-B4 cells (ATCC) were kindly provided by Dr. Susan Fagan (University of Georgia), were cultured in DMEM high glucose medium (Gibco) and 10% FBS (Atlantic biologics), 100 I.U./mL penicillin, and 100 (μg/mL) streptomycin (ATCC). Cells were cultured at a density of 15,000 cells/well in a chamber slide (Thermo Fisher) with complete medium and treated with 25 μg/mL acrolein-BSA conjugate (StressMarq Biosciences) in complete medium for 6 h. Following the incubation, the culture media was removed, and cells were washed with 1× PBS and fixed with 4% paraformaldehyde for 10 min and subjected to immunocytochemistry according to the method standardized in our laboratory [35,96]. This was followed by PBS wash, and the chamber slides were stored in humidified containers at 4 °C. Permeabilization of the cells was performed using 0.1% Triton X-100 in PBS (5 min), followed by PBS wash, and blocking was achieved using 10% donkey serum at room temperature for 1 h. Cells were washed and incubated with respective primary antibodies (Table 1) overnight. The next day, the cells were washed with PBS and incubated with appropriate secondary antibodies for 2 h. The chamber slides were washed, and the chambers separated from the glass slide. Cells were covered with a coverslip using a mounting medium containing DAPI and stored at 4 °C. Images were taken using a confocal microscope (LSM 780; Carl Zeiss, Thornwood, NY, USA) available at the Augusta University imaging core facility.

### 4.9. Measurement of ROS Generation Using CM-H2DCFDA Assay

This assay was performed using CM-H2DCFDA (C6827, Thermo Fisher) following the manufacturer’s protocol. After appropriate treatment, C8-B4 cells were washed with pre-warmed PBS and incubated with ROS-sensitive probe CM-H2DCFDA (40 μM) at room temperature in the dark for 30 min. Cells were washed carefully with warm PBS and covered with a mounting medium with DAPI (Vectashield; Vector Laboratories, Burlingame, CA, USA). Images were taken immediately by using the FITC channel. The intensity was quantified by using NIH ImageJ software v1.53k.

### 4.10. Data Analysis

GraphPad Prism 9 was used for statistical analysis. Results are presented as mean ± SD. Statistical analysis was performed using one-way ANOVA followed by the Turkey test for multiple comparisons. The Student’s test was used in case of a single comparison.

## Figures and Tables

**Table 1:** List of antibodies used in the study.

Antibody	Catalog Number	Source	Dilution	Experiment
Iba-1	019-19741	Wako	1:500	Immunostaining
Iba-1	17198	Cell Signaling Technology	1:500	Western blot
Arginase-1	Abs535	EMD Millipore	1:200	Immunostaining
CD68	ab125212	Abcam	1:500	Immunostaining
CD16/32	553142	BD Biosciences	1:500	Immunostaining
CD206	ab64693	Abcam	1:500	Immunostaining
F4/80	ab6640	Abcam	1:100	Immunostaining
Nrf2	12721	Cell Signaling Technology	1:1000	Western blot
HO-1	43966	Cell Signaling Technology	1:1000	Western blot
Conjugated-acrolein	ab48501	Abcam	1:1000	Western blot
β-Actin	A1978	Sigma-Aldrich	1:10000	Western blot

**Table 2:** Primer sequences used in the study.

Gene Name	Forward Primer	Reverse Primer
TNFα	GGTCCCCAAAGGGATGAGAA	TGAGGGTCTGGGCCATAGAA
IL1β	CCAAGCAACGACAAAATACC	GTTGAAGACAAACCGTTTTTCC
IL10	GCTCTTACTGACTGGCATGAG	CGCAGCTCTAGGAGCATGTG
MCP-1	GGCTCAGCCAGATGCAGTTAA	CCTACTCATTGGGATCATCTTGCT
IL6	AGACAAAGCCAGAGTCCTTCAG	TGCCGAGTAGATCTCAAAGTGA
IL18	TCAAAGTGCCAGTGAACCCC	GGTCACAGCCAGTCCTCTTAC
COX2	AAGCCAACATGATTGTTGTGAA	CGGCAGCAGTCACATACTTA
IL21	ATCCTGAACTTCTATCAGCTCCAC	GCATTTAGCTATGTGCTTCTGTTTC
CCL5	GCTGCTTTGCCTACCTCTCC	TCGAGTGACAAACACGACTGC
TGFβ	CCCGAAGCGGACTACTATGC	ATAGATGGCGTTGTTGCGGT
IL12	CTCAGGATCGCTATTACAATTCCTC	TTCCAACGTTGCATCCTAGGATC
IL17	CTCCAGAAGGCCCTCAGACTAC	AGCTTTCCCTCCGCATTGACACAG
CCL3	TTCTCTGTACCATGACACTCTGC	CGTGGAATCTTCCGGCTGTAG
IL4	GGTCTCAACCCCCAGCTAGT	GCCGATGATCTCTCTCAAGTGAT
IL13	CCTGGCTCTTGCTTGCCTT	GGTCTTGTGTGATGTTGCTCA
HPRT	GAAAGACTTGCTCGAGATGTCATG	CACACAGAGGGCCACAATGT
